# GD-DAMNet: Real-Time UAV-Based Overhead Power-Line Presence Recognition Using a Lightweight Knowledge Distillation with Mamba-GhostNet v2 and Dual-Attention

**DOI:** 10.3390/e28020166

**Published:** 2026-01-31

**Authors:** Shuyu Sun, Yingnan Xiao, Gaoping Li, Yuyan Wang, Ying Tan, Jundong Xie, Yifan Liu

**Affiliations:** 1 College of Engineering & Technical, Chengdu University of Technology, Leshan 614000, China; shuyu_sun48@163.com (S.S.); yingnan_xiao@163.com (Y.X.); 2 School of Mathematics, Southwest Minzu University, Chengdu 610041, China; ty7499@swun.edu.cn; 3 College of Computer Science and Artificial Intelligence, Southwest Minzu University, Chengdu 610041, China; 240835002003@stu.swun.edu.cn (Y.W.); 240854042026@stu.swun.edu.cn (J.X.); 230835002029@stu.swun.edu.cn (Y.L.)

**Keywords:** unmanned aerial vehicle (UAV), deep neural network, dual-attention mechanism, real-time power-line presence recognition, mobile devices

## Abstract

Power-line presence recognition technology for unmanned aerial vehicles (UAVs) is one of the key research directions in the field of UAV remote sensing. With the rapid development of UAV technology, the application of UAVs in various fields has become increasingly widespread. However, when flying in urban and rural areas, UAVs often face the danger of obstacles such as power lines, posing challenges to flight safety and stability. To address this issue, this study proposes a novel method for presence recognition in UAVs for power lines using an improved GhostNet v2 knowledge distillation dual-attention mechanism convolutional neural network. The construction of a real-time UAV power-line presence recognition system involves three aspects: dataset acquisition, a novel network model, and real-time presence recognition. First, by cleaning, enhancing, and segmenting the power-line data collected by UAVs, a UAV power-line presence recognition image dataset is obtained. Second, through comparative experiments with multi-attention modules, the dual-attention mechanism is selected to construct the CNN, and the UAV real-time power-line presence recognition training is conducted using the SGD optimiser and Hard-Swish activation function. Finally, knowledge distillation is employed to transfer the knowledge from the dual-attention mechanism-based CNN to the nonlinear function and Mamba-enhanced GhostNet v2 network, thereby reducing the model’s parameter count and achieving real-time recognition performance suitable for mobile device deployment. Experiments demonstrate that the UAV-based real-time power-line presence recognition method proposed in this paper achieves real-time recognition accuracy rates of over 91.4% across all regions, providing a technical foundation for advancing the development and progress of UAV-based real-time power-line presence recognition.

## 1. Introduction

The presence recognition of power lines is crucial for maintaining the reliability of modern electrical grids. In this context, Unmanned Aerial Vehicle (UAV) real-time power-line presence recognition has emerged as a pivotal technical method that utilises UAV technology to inspect power lines on the ground [1,2]. Modern power facility upgrades have driven a significant expansion in the scale of power grids, with coverage density increasing by over 300% compared to a decade ago [3]. The reliability of power systems is critical to modern society, directly impacting economic development and social stability [4]. Traditional recognition methods (manual patrols, temperature/acoustic monitoring) suffer from issues such as poor real-time performance, high costs, and significant safety hazards [5], making it difficult for them to satisfy the maintenance requirements of complex power grids. First, manual patrols require substantial human and material resources, are time-consuming and labour-intensive, and have low efficiency [6,7]; secondly, manual recognitions pose certain safety risks, especially in complex terrain or adverse weather conditions, where personnel safety cannot be effectively guaranteed [8]; furthermore, traditional methods often fail to cover power lines in remote or complex areas, resulting in recognition blind spots [9]. Therefore, seeking an efficient, safe, and intelligent power-line presence recognition method has become an urgent need [10]. The widespread application of UAV technology has enabled it to play a significant role in fields such as agriculture, environmental monitoring, and search and rescue [11]. UAVs possess flexible manoeuvrability and the advantage of high-altitude overview, enabling them to quickly reach target areas and carry various sensors and equipment to achieve efficient recognition of ground targets [12]. This not only avoids the safety risks associated with personnel contact with wires but also enables rapid and effective recognition of wires over a larger area [13]. Additionally, UAVs possess autonomous and intelligent characteristics [14]. Computer vision technology integrated with deep learning algorithms can perform real-time analysis of aerial imagery, enabling autonomous identification of power-line targets and establishing a closed-loop intelligent inspection system from data collection to defect diagnosis [15].

In recent years, with the development of artificial intelligence technology, deep learning-based power system recognition technology has emerged rapidly and demonstrated significant potential in improving detection accuracy and real-time performance. Convolutional Neural Networks (CNNs) are one of the most widely used model architectures, among which algorithms such as YOLO (You Only Look Once) [16,17] and Faster Region-Based Convolutional Neural Network (R-CNN) [18] are widely used for real-time recognition of obstacles around UAVs. Chen et al. [19] developed a UAV inspection video parsing model that integrates YOLO with a CNN architecture, achieving automated counting of tower components by the real-time capture of critical nodes in distribution networks. Validation results indicate that this method can facilitate rapid disaster damage assessment and support optimised emergency repair decision-making. Zhao et al. [20] constructed a power-line dataset graded by image clutter, proposing a dual-stream algorithm that integrates detection and semantic segmentation. Cross-scenario testing shows that this method has better adaptability than traditional methods, providing new insights for UAV inspections, but attention should be paid to flight safety risks in urban and rural environments. Li et al. [21] developed a lightweight UAV inspection system that uses “image processing-millimetre wave radar fusion” technology to achieve autonomous inspection of overhead ground wires. It obtains real-time position information through traditional image processing, combines radar data to complete pixel-to-actual-distance error conversion, and uses flight path correction to stabilise the ground wire at the centre of the video, providing a lightweight solution for automated monitoring of urban power transmission lines. Damodaran et al. [22] constructed a four-level detection framework comprising “pre-processing-DL model-classification algorithm-Hoff transform” achieving high-precision power-line identification in complex aerial photography scenarios, with a 40% improvement in detection efficiency, providing a new paradigm for multi-modal fusion. Mao et al. [23] developed a ±500 kV DC line electromagnetic field simulation model to address safety protection requirements, revealing the distribution patterns of electromagnetic fields and quantitatively assessing safety thresholds. They established a safety operation parameter database for multi-rotor UAVs, providing a dynamic protection benchmark for intelligent recognition of ultra-high-voltage lines. Existing CNN networks suffer from insufficient accuracy and low computational efficiency when recognising small, complex wire targets, and they still have limitations in terms of real-time performance and generalisation in complex scenarios. There is an urgent need to develop high-precision, lightweight, and efficient recognition technologies to ensure the safety of UAV power-line recognition operations. To address these issues, this paper proposes a novel deep neural network-based UAV power-line recognition method using an improved GhostNet v2 [24] knowledge distillation dual-attention mechanism (DAM) based on Mamba [25], designed for real-time high-precision recognition of overhead power lines in the field. This study aims to enhance performance in real-time power-line presence recognition tasks by introducing the DAM. The DAM can more precisely focus on the distinctive features of the small-scale target (power lines) during feature extraction, enhancing the network’s adaptability to complex scenarios. Subsequently, the knowledge distillation method based on Mamba-enhanced GhostNet v2 is applied to the aforementioned convolutional deep neural network to achieve knowledge distillation, thereby lightweighting the power-line recognition network and enabling real-time presence recognition. This strategy maintains high accuracy while improving the model’s real-time performance, thereby providing a more efficient and reliable solution for power-line recognition in power systems. The main contributions of this paper are as follows:

(1) Establish a training dataset for UAV power-line presence recognition. By cleaning, enhancing, and partitioning the UAV image data, we form a power-line presence recognition dataset suitable for the proposed network for training and learning.

(2) Propose a novel DAM that integrates the Convolutional Block Attention Module (CBAM) [26] and the Large Separative Kernel Attention module (LSKA) [27] to construct a deep neural network for power-line presence recognition. Meanwhile, we improve the GhostNet v2 network by using nonlinear functions and Mamba blocks, replace the linear transformations in the network with nonlinear transformations, and replace the ordinary Convolution with Depthwise Separable Convolution [28] Meanwhile, the Mamba block module is added to improve the recognition accuracy.

(3) By adopting the method of knowledge distillation, the knowledge information of the proposed DAM network is transferred to the lightweight GhostNet v2 network improved by Mamba, thereby reducing the number of parameters and training time of the model to adapt to the construction of mobile devices.

This paper introduces a DAM and Mamba block module to enhance the model’s feature extraction capabilities, particularly in complex backgrounds. Through experiments on multiple video power-line presence recognition datasets, this paper demonstrates the advantages of the improved model in terms of accuracy and real-time performance. The proposed network structure is highly scalable and can be applied to a wider range of power system recognition tasks.

## 2. Dataset and Image Preprocessing

Building a UAV power-line presence recognition dataset is a complex and systematic process involving multiple aspects such as data collection, annotation, processing, and management. The successful implementation of this process is critical for the development of deep learning-based power-line monitoring and maintenance systems. This paper will provide a detailed discussion on how to establish a high-quality dataset for UAV power-line presence recognition, including hardware selection, data collection workflow, data preprocessing, annotation standards and formats, data management, and solutions.

### 2.1. UAV Models and Equipment Configurations

As the main tool for aerial data collection, the performance of UAVs directly affects the quality of the dataset. Different types of UAVs have different flight stability, endurance, and load capacity. In this study, the DJI (Shenzhen Dajiang Innovation Technology Co., Ltd, Shenzhen, China) Air 2S UAV is selected, and its specific performance is as follows:

1. Battery capacity and battery life: The DJI Air 2S comes with a 3500 mAh Smart Flight Battery, with a maximum flight time of 31 min (windless environment, stable speed).

2. Flight Stability: The DJI Air 2S is equipped with the advanced APAS 4.0 Advanced Flight Assist System, which is able to avoid obstacles intelligently and realise smooth flight. It supports four-direction obstacle avoidance to avoid collision; it can withstand level 5 wind (8.5–10.5 m/s), showing excellent wind resistance; and the maximum speed in sport mode is up to 68.4 km/h.

3. Load capacity: The DJI Air 2S weighs in at 595 g, in keeping with its lightweight design; while this drone is not specifically designed to carry other payloads, its primary mission is aerial photography and cinematography.

4. Imaging Performance: The DJI Air 2S is equipped with a 1-inch CMOS sensor to capture more details and improve the quality of photos in low light conditions. Up to 20 MP still photos and 5.4K/30 fps and 4K/60 fps video recording are supported, and the images are very clear and rich in detail. Using the OcuSync 3.0 image transmission system, it supports HD video transmission up to 12 km to ensure smooth real-time picture during long distance flight.

The DJI Air 2S UAV combines long endurance, strong flight stability, advanced obstacle avoidance features, and high-resolution imaging sensors, making it a suitable UAV for taking high-quality pictures and videos.

### 2.2. Data Acquisition

Data acquisition is a core aspect of building a dataset for UAV power-line presence recognition. Collecting high-quality image and video data can provide a solid foundation for subsequent annotation and model training. Typically, data acquisition is divided into two parts:

(1) Image acquisition: UAVs fly along power lines to capture high-resolution images of the power lines and their surroundings. These images may include multiple angles of the power lines to ensure that all possible fault points are covered. The acquisition is performed at different times of day (e.g., daytime, dusk) and in different weather conditions (e.g., sunny, cloudy, foggy) to increase the diversity of the dataset.

(2) Video acquisition: Video data can help capture dynamic changes in power lines, such as power lines swinging, birds touching power lines, etc. By analysing the video data, it is possible to effectively detect the location of power lines.

High wind speed or bad weather may affect the flight stability of the UAV and the clarity of image acquisition, so it is challenging to perform acquisition in bad weather. The power lines may be obscured by trees when flying in mountainous areas or dense forests, resulting in the loss of power lines in the image, which requires more accurate flight control. Due to the vibration of the UAV in flight, light changes, etc., the captured images may be blurred or noisy, and these images may need to be filtered or denoised in post-processing. The specific collection time, location, flight altitude, weather conditions, and other information are shown in Table 1:

### 2.3. Image Preprocessing

In this study, the training dataset requires images in JPEG or PNG format; therefore, all other formats are converted accordingly. Low-quality samples—such as overly blurred images, images with excessive noise, or corrupted video clips—are removed to maintain dataset integrity. Ensuring high data quality is crucial for improving model performance. To enhance the model’s robustness, data augmentation techniques are applied to perform necessary data augmentation operations on images, such as image flipping, rotation, brightness adjustment, cropping, and other operations. Through data enhancement, the number of samples in the dataset can be expanded effectively and the overfitting of the model can be reduced.

**Figure 1:**
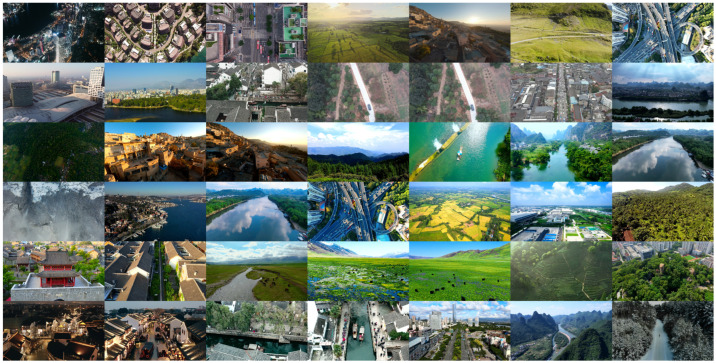
Partial images from the power-line-free dataset.

**Figure 2:**
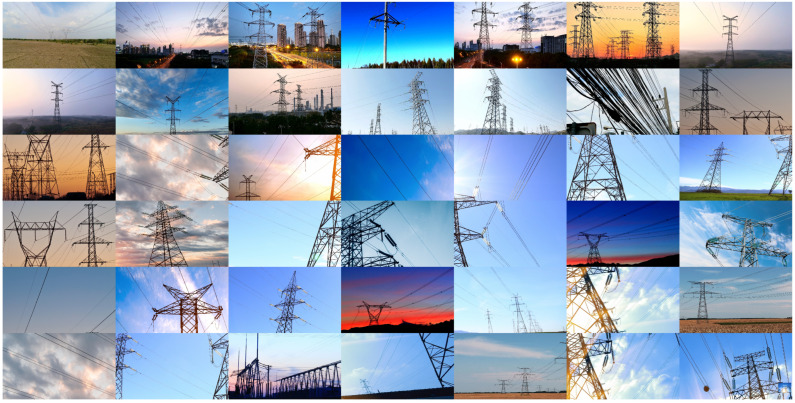
Partial images from the power-line dataset.

Following labelling and preprocessing, the dataset needs to be reasonably divided to ensure the effectiveness of model training. The dataset of this paper is divided according to the following proportions: Train set, which accounts for 80% of the total data (1162 images without power lines, 1236 images with power lines), is used to train the deep neural network model; Validation set, which accounts for 10% of the total data (100 images without power lines, 100 images with power lines), is used for the validation of the model in the process of training to adjust the hyper-parameters; Test set, which accounts for 10% of the total data (100 images without power lines, 100 images with power lines), is used for the final evaluation of the model. In order to ensure that the model can be generalised to more scenarios, attention should be paid to maintain as much data diversity as possible when dividing the dataset, such as different power-line angles and different time periods of data collection. which should be evenly distributed in the Train set, Validation set, and Test set. Representative samples from the training set are illustrated in Figure 1 and Figure 2:

With the development of UAV technology and artificial intelligence technology, the way of constructing datasets for power-line recognition is also evolving. In addition, with the popularisation of 5G technology, real-time remote monitoring and data uploading will further promote the application of UAV power-line recognition. Building a UAV power-line recognition dataset is a systematic project that involves multiple technologies and links and requires meticulous planning and execution. The construction of high-quality datasets will promote the intelligent development of power-line recognising.

## 3. Building CNNs Based on Dual-Attention Mechanism

Although various neural networks have demonstrated excellent performance in visual tasks, they still face challenges such as insufficient feature extraction and severe background interference when recognising small objects (e.g., power lines). To enhance the model’s ability to recognise wires, this paper introduces the DAM, which combines the CBAM and LSKA attention modules to enhance feature expression across channels, spatial dimensions, and multiple scales, thereby improving the model’s ability to perceive details in the target area.

### 3.1. DAM Constructs a CNN Framework

The DAM improves the recognition performance of slender targets by learning channel features and enhancing the response of key features. This mechanism effectively suppresses background interference through spatial multi-scale weighting and channel adaptive adjustment. The specific design, module structure, and training steps of the DAMNet network are detailed in Table 2.

**Figure 3:**
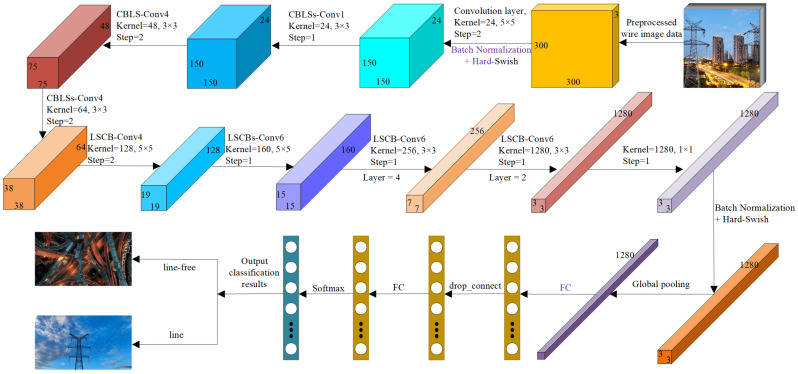
CNN framework structure based on DAM.

The network proposed in this paper is constructed based on a DAM, incorporating a progressive learning strategy and an adaptive regularisation strength adjustment mechanism to accelerate training speed and further enhance model performance. The main framework of the CNN model constructed based on the DAM is shown in Figure 3:

**1-stage CBLSConv1, k 3×3 module structure**

**Figure 4:**
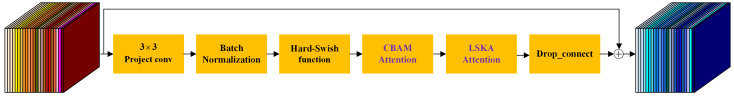
1-Stage CBLSs-Conv1 module structure diagram.

This module follows the following steps: first, the convolved low-dimensional feature maps are input; second, the input low-dimensional feature maps are convolved for feature extraction; third, the upsampled feature maps are subjected to batch normalisation (BN) [29] and Hard-Swish activation processing [30]; fourth, the activated feature maps are enhanced using the CBAM attention mechanism [31]; fifth, the enhanced feature maps are processed using the LSKA attention mechanism to capture long-range dependencies in the image; finally, the enhanced feature maps are subjected to Drop_connect [32] and added to the initial feature maps (i.e., Residual Connection). The structure of the CBLSs-Conv1 module is shown in Figure 4:

**2-stage and 3-stage: CBLSsConv4, k 3×3 module structure and CBLSConv4, k 3×3 module structure**

The 2-stage process is as follows: first, input the feature maps processed by 2-stage into the feature extraction module; second, expand the channel dimension of the input low-dimensional feature maps through 3×3 convolution (dimension expansion, this step expands the number of channels of the feature maps obtained by 2-stage by 4 times, with a stride of 2); third, perform BN and Hard-Swish activation processing on the dimension-expanded feature maps; fourth, apply the CBAM attention mechanism to the activated feature maps for feature enhancement; fifth, the feature-enhanced feature maps are processed using the LSKA attention mechanism to capture long-range dependencies in the image; sixth, the feature-enhanced feature maps are downsampled using a 1×1 convolution to project them back to the channel dimension of the initial input feature maps (the channel dimension obtained after the 2-stage processing); finally, the downsampled feature maps are subjected to BN processing once again.

**Figure 5:**
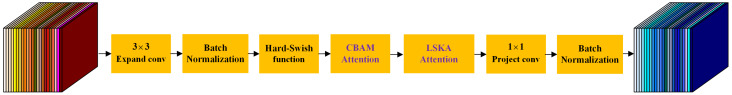
2-Stage CBLS-Conv4 module structure diagram.

**Figure 6:**
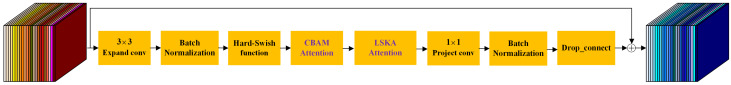
3-Stage CBLSs-Conv4 module structure diagram.

The 3-stage process is similar to the 2-stage process, except that in the final part, the enhanced feature map is subjected to Drop_connect and added to the initial feature map to obtain the final feature map. The structures of the CBLSsConv4 and CBLSConv4 modules are shown in Figure 5 and Figure 6:

**4-stage: LSCB-Conv4, k 5×5 module structure**

**Figure 7:**
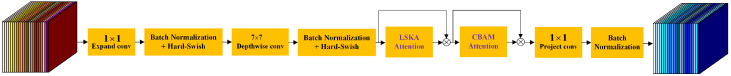
4-Stage LSCB-Conv4 module structure diagram.

The specific steps of the 4-stage module are as follows: first, input the feature maps processed by the 3-stage module into the module for feature extraction; second, expand the channel dimension of the input low-dimensional feature maps through a 1×1 convolution (dimension expansion, this step expands the number of channels of the feature maps obtained by the 3-stage module by 4 times); third, perform BN and Hard-Swish activation on the dimension-expanded feature maps; fourth, use a 5×5 depthwise separable convolution (DSC) with a stride of 2 to extract features from the activated feature maps; fifth, perform BN and Hard-Swish activation on the feature maps after feature extraction; sixth, feature enhancement is performed using the LSKA attention mechanism; seventh, the feature-enhanced feature map is scaled and added to the feature map obtained in the fifth step; eighth, feature enhancement is performed on the activated feature map using the CBAM attention mechanism; ninth, the enhanced feature map is added to the feature map obtained in the sixth step; tenth, the added feature map is subjected to a 1×1 convolution for dimension reduction and projected onto the channel dimension of the feature map obtained from the input 3-stage; finally, the dimension-reduced feature map is subjected to BN processing and output. The structure of the LSCB-Conv4 module is shown in Figure 7:

**5, 6 and 7-stage: LSCBs-Conv6, k 5×5; and LSCB-Conv6, k 3×3 module structure**

**Figure 8:**
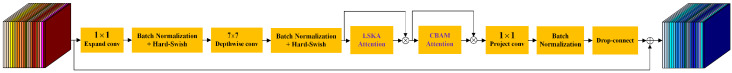
5-Stage LSCBs-Conv6 module structure diagram.

**Figure 9:**
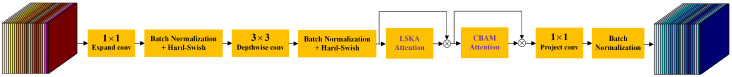
6, 7-Stage LSCB-Conv6 module structure diagram.

The steps of the 5-stage module are similar to those of the 4-stage module, with the following specific differences: second, the low-dimensional feature maps are expanded in channel dimension (up-sampled) through a 1×1 convolution (this step expands the number of feature map channels obtained in the 4-stage module by a factor of 6); fourth, perform feature extraction on the activated feature maps using a 5×5 depthwise separable convolution (step size = 1); finally, apply a Drop_connect operation to the batch-normalised feature maps and add them to the initial input feature maps from the first step (i.e., Residual Connection). The steps for the 6 and 7-stage modules are almost identical to those of the 4-stage module, with the exception that in the fourth step, the activated feature maps are processed using a 3×3 depthwise separable convolution for feature extraction, with a stride of 1. The module structures of LSCBs-Conv6 and LSCB-Conv6 are shown in Figure 8 and Figure 9:

**8-Stage: Conv 1×1 and Pooling and FC**

The final step of the proposed network consists of three components: a convolutional layer (Conv), a pooling layer (Pooling), and a fully connected layer (FC). Specifically:

Conv (Convolution): This step is a 1×1 convolution layer used to adjust the number of channels in the feature map to match the subsequent fully connected layer (FC). This convolution layer may also include BN and an activation function (Swish activation).

Pooling: The pooling layer is used to reduce the dimensions of the feature map (i.e., height and width) while preserving important feature information. This paper uses the Global Average Pooling (GAP) strategy, which converts the entire feature map into a single vector, where each element is the average value of the corresponding channel.

FC (Fully Connected): The fully connected layer is used to convert the feature map into the final output. In classification tasks, the number of neurons in the output layer typically equals the number of categories. The fully connected layer also includes dropout operations to reduce the risk of overfitting.

### 3.2. Optimiser Design

In this section, Stochastic Gradient Descent (SGD) is used as the optimiser [33]. The algorithm minimises J(θ) by updating θ along the opposite direction of the gradient $\nabla_{\theta }J\left(\theta \right)$, given the objective function J(θ) and the model parameters $\theta \in R^{d}$ to be optimised. The process of gradient descent involves the following steps:

Assume that there are *n* training samples, denoted as $\left\{\left(x_{1},y_{1}\right),\left(x_{2},y_{2}\right),\cdots ,\left(x_{n},y_{n}\right)\right\}$, and for the *i*th pair of samples $\left(x_{i},y_{i}\right)$, the gradient of the loss function with respect to the model parameters is calculated as $\nabla_{\theta }J_{i}\left(\theta ,x^{i},y^{i}\right)$.

The learning rate is set to α, and the parameters are updated by the Stochastic Gradient Descent (SGD) method, following Equation (1):

(1) $$\theta_{i+1}=\theta_{i}-a\cdot \nabla_{\theta }J_{i}\left(\theta ,x^{i},y^{i}\right)$$

The primary advantage of SGD lies in its ability to calculate the gradient of a single data sample in each iteration, thereby improving the efficiency of parameter updates. The deep learning model adopted in this study integrates a DAM with an SGD (stochastic gradient descent) optimiser, offering the following advantages:

Compared to the Adam optimiser [34], the SGD optimiser offers more intuitive parameter adjustments, helping the network converge faster and improve classification task accuracy. By introducing a dual-attention structure into the network based on deep separable convolutions, this improvement enhances network performance and effectively shortens the training cycle.

### 3.3. Knowledge Distillation

Knowledge Distillation (KD) [35] is a technique of deep learning model compression, which aims to maintain the performance of a model while reducing the consumption of computational resources by transferring the knowledge from a large, complex model (often referred to as the teacher model) to a smaller model (often referred to as the student model). This approach has gained a lot of attention in the field of deep learning in recent years, especially when deploying deep learning models on resource-constrained devices (e.g., mobile devices, embedded devices), where it plays a crucial role. Additionally, the training and testing of deep learning models can be time-consuming, especially when the model has a large number of layers, which significantly increases inference time. For example, language models based on the Transformer architecture have achieved very high accuracy in various natural language processing tasks, but these models have long training and inference times and consume a significant amount of memory [36]. To address these challenges, researchers have proposed various model compression methods, including weight pruning, quantisation, low-rank decomposition, and knowledge distillation. Among these, knowledge distillation is considered one of the most effective methods, as it can compress the model while preserving its performance. This paper improves upon GhostNet v2, and we refer to it as MambaDN-GhostNet. Its framework structure primarily consists of the following components:

(1) The core module of GhostNet is the Ghost Module [37], which generates features through linear transformations to reduce redundant computations but has limited expressive power. This study introduces nonlinear operations in GhostNet v2 to enhance feature expression.

(2) Standard convolutions are replaced with depthwise separable convolutions, i.e., depthwise convolutions and pointwise convolutions, effectively reducing computational and parameter costs. In GhostNet v2, this convolution is combined with the Ghost Module to improve efficiency and feature extraction capabilities.

(3) Integrating the Mamba block lightweight attention mechanism into GhostNet v2 to weight feature channels, enhancing the model’s ability to perceive key features and improving accuracy and robustness.

(4) The improved GhostNet v2 adopts a modular design, supporting flexible combination and replacement of functional modules to enhance the network’s scalability and adaptability. This design endows the network with strong scalability and adaptability, enabling it to address future complex application requirements.

**Figure 10:**
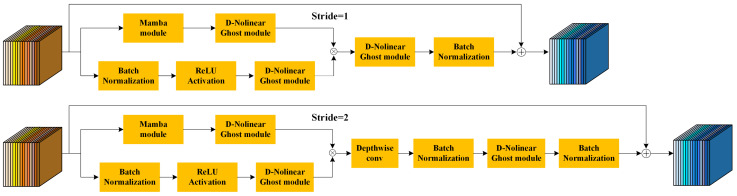
MambaDN-GhostNet network structure.

**Figure 11:**
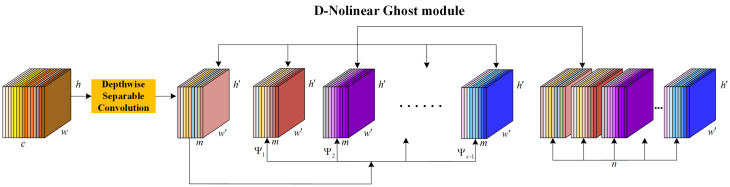
D-Nolinear Ghost module network structure diagram.

The improved GhostNet v2 aims to maintain lightweight architecture while enhancing performance in tasks such as image classification, object detection, and semantic segmentation through structural optimisation and technical improvements. The specific network structure of GhostNet v2 is shown in Figure 10:

The process of generating D-Nolinear Ghost feature maps uses a nonlinear operation Ψ for mapping instead of the original linear operation. Specifically, as shown in Figure 11:

D-Nolinear Ghost module network module: First, perform a separable convolution operation on the input feature map to generate an intrinsic feature map with *m* channels and dimensions $h^{\prime }\times w^{\prime }$. Next, apply a nonlinear transformation $\Psi_{i}$, i=1,2,…,s−1 to the intrinsic feature map to generate s−1 sets of D-Nolinear Ghost maps. Combine the intrinsic feature map and the s−1 sets of D-Nolinear Ghost maps as the output, resulting in n=s×m channels of D-Nolinear Ghost maps with dimensions $h^{\prime }\times w^{\prime }\times n$.

**Figure 12:**
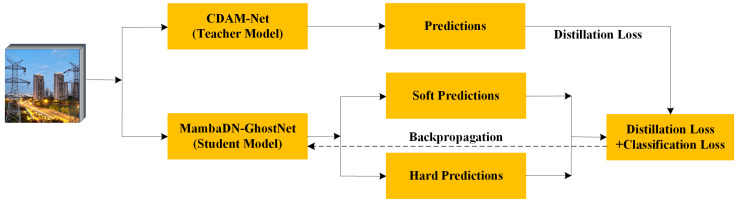
Relationship-based knowledge distillation structure diagram.

The MambaDN-GhostNet network achieves architectural optimisation to reduce computational and storage requirements while striving to maintain or improve model accuracy. This is of significant importance for the application of deep learning models on mobile devices, embedded systems, and IoT terminals. The framework structure of knowledge distillation is as follows Figure 12:

Teacher Model: This paper uses the proposed DAM-Net network model as the teacher model. The probability distribution output by the DAM-Net network model in the knowledge distillation framework is a ’soft’ label, which contains richer inter-category mutual information.

Student Model: In this paper, we use MambaDN-GhostNet as the student model because it has far fewer parameters than the proposed DAM-Net network model. The goal of MambaDN-GhostNet is to learn from the teacher model to produce outputs that are as close as possible to those of the teacher model.

The core idea of knowledge distillation is to transfer knowledge from a complex teacher model to a smaller student model to improve the performance of the student model. Knowledge distillation involves multiple mathematical formulas to achieve this knowledge transfer. The following introduces the key formulas related to it and their explanations.

(1) Softmax with Temperature Scaling

In Knowledge Distillation, the output of a teacher model is usually passed through a Softmax function to generate a probability distribution. However, to allow the student model to learn from the teacher model, a temperature parameter *T* is introduced, which is used to smooth the output distribution of the teacher model. The Softmax function is usually defined as Equation (2):

(2) $$\sigma \left(z_{i}\right)=\frac{e^{z_{i}}}{\sum_{j=1}^{n}e^{z_{j}}}$$

For an input vector $z=[z_{1},z_{2},...,z_{n}]$, where $z_{i}$ is the logits of class *i*. For the introduction of the temperature parameter *T*, the Softmax function becomes Equation (3):

(3) $$\sigma_{T}\left(z_{i}\right)=\frac{e^{z_{i}/T}}{\sum_{j=1}^{n}e^{z_{j}/T}}$$

When T>1, the distribution becomes smoother, and the student model can better learn the knowledge hidden in the teacher model.

When T=1, the Softmax function is the standard Softmax.

When T<1, the model will favour the large probability output.

In knowledge distillation, a higher temperature T<1 is typically used to extract soft targets from the teacher model, allowing the student model to learn richer category information.

(2) Loss function of Knowledge Distillation

In Knowledge Distillation, the training of student models is guided by a loss function that consists of two main components:

(1) Distillation Loss: measures the difference between the soft-labelled output of the student model and the teacher model.

(2) Classification Loss: measures the difference between the output of the student model and the true labels.

One is the distillation loss between the student model and the real labels (cross-entropy loss), and the other is the distillation loss between the student model and the teacher model output (Kullback–Leibler Divergence). The overall loss function can be expressed as Equation (4):

(4) $$L=\left(1-\alpha \right)L_{CE}\left(y,{\hat{y}}_{S}\right)+\alpha \cdot T^{2}\cdot L_{KL}\left(p_{T}^{T},p_{T}^{S}\right)$$

where $L_{CE}$ is the standard cross-entropy loss function used to compare the difference between the output ${\hat{y}}_{S}$ of the student model and the true label *y*. $L_{KL}$ is the Kullback–Leibler Divergence (KL Divergence), which measures the difference between the soft target $p_{T}^{T}$ of the teacher model and the soft target $p_{T}^{S}$ of the student model. *T* is the temperature parameter, used to smooth the probability distribution. α is a weight parameter used to balance the relative importance of classification loss and distillation loss.

(1) Cross Entropy Loss: the Cross Entropy Loss Function $L_{CE}$ is commonly used for multi-category classification tasks and is formulated as follows in Equation (5):

(5) $$L_{CE}\left(y,{\hat{y}}_{s}\right)=-\sum_{i=1}^{n}y_{i}\log\left({\hat{y}}_{S},i\right)$$

where $y_{i}$ is the true label, ${\hat{y}}_{S},i$ is the predicted probability of the student model for the category *i*.

(2) Kullback–Leibler Divergence Loss: KL divergence is used to measure the difference between two probability distributions and is used in knowledge distillation to compare the output probability distributions of the teacher model with those of the student model. The formula for the KL divergence is Equation (6):

(6) $$L_{KL}\left(p_{T}^{T},p_{T}^{S}\right)=\sum_{i=1}^{n}p_{T}^{T}\left(i\right)\log\left(\frac{p_{T}^{T}\left(i\right)}{p_{T}^{S}\left(i\right)}\right)$$

where $p_{T}^{T}\left(i\right)$ is the predicted probability of the teacher model for category *i* and $p_{T}^{S}\left(i\right)$ is the predicted probability of the student model for category *i*. The goal of KL divergence is to minimise the difference in probability distributions between the student model and the teacher model.

The training process for overall knowledge distillation is as follows: first, train a complex model (DAM-Net), which typically has high performance but a large number of parameters and high computational complexity; construct a smaller student model (MambaDN-GhostNet), which has fewer parameters and a relatively simple structure. The teacher model’s predictions for the input data are passed to the student model as “soft labels”; Train the student model by minimising the loss function (including classification loss and distillation loss), aiming to make its outputs as consistent as possible with those of the teacher model; after multiple iterations of training, the student model learns to mimic the teacher model and ultimately completes knowledge distillation.

(3) Role of the weight parameter α: The weight parameter α controls the trade-off between classification loss and distillation loss. When α is close to 1, the student model relies more on the teacher model for learning, and the role of distillation loss is dominant. When α is close to 0, the student model relies more on the true labels, the influence of distillation loss is weakened, and the training process is closer to traditional supervised learning.

In this section, the DAM-Net network is used as the teacher model and the MambaDN-GhostNet is used as the student model. By using the output of the DAM-Net network model as soft labels and passing them to the student model for training, the knowledge of the teacher model is leveraged to guide the training process of the student model. By optimising the parameters to gradually approach the prediction results of the DAM-Net network model, the performance of the student model can be improved. During the distillation process, adjustments and optimisations are made to achieve optimal performance and convergence. By transferring knowledge from the teacher model to the student model, knowledge distillation not only significantly reduces the number of model parameters and computational overhead but also enhances the generalisation ability and performance of small models in many cases.

## 4. Experimental Results and Analysis

### 4.1. Experimental Setup

To validate the effectiveness of the proposed GD-DAMNet network in the real-time presence recognition task of power lines, this study designs a series of experiments to evaluate the performance of the model. The experiments are conducted on a server equipped with an NVIDIA RTX 3090 GPU, with Windows 11 as the operating system, and with PyTorch 2.0 as the deep learning framework. Mixed-precision training was employed to improve training efficiency and reduce memory usage. The SGD optimiser is used, with the initial value of the learning rate set to 0.001 and the weight decay factor set to 1 × 10^−4^. The loss function is the cross entropy loss function. The batch size was set to 25 to balance computational efficiency and memory requirements. The model is trained for 20 epochs, and the learning rate is dynamically adjusted by using the CosineAnnealingLR scheduling strategy.

### 4.2. Dataset Training Results and Analysis

**Figure 13:**
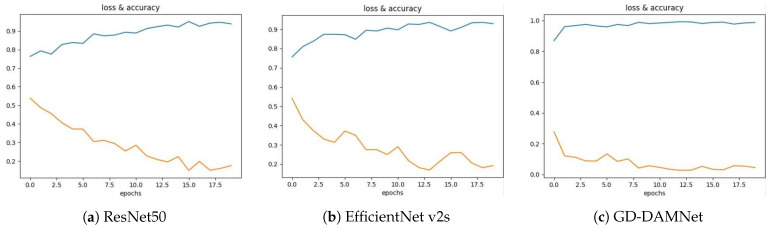
The loss function value (loss) and the accuracy of the training process with the number of training rounds (epochs).

In this study, we demonstrate the practical situation of three different neural network models, ResNet50 [38], EfficientNet v2s [39], and GD-DAMNet, with respect to the loss function value (loss) and accuracy with the number of training rounds (epochs) during the training process. The details are shown in Figure 13:

The training results of each model are divided into three graphs: ResNet50, EfficientNet v2s, and GD-DAMNet. The horizontal axis of each graph indicates the number of training rounds (epochs), and the vertical axis indicates the accuracy (blue curve) and the loss function value (orange curve), respectively. In the following, we will analyse the training rounds, accuracy, loss function value, and training stability in detail.

As shown by the horizontal axis in each graph, all models were trained for 20 epochs. Throughout these epochs, we can observe the trend of accuracy improvement and loss function decline of each model. By observing the blue accuracy curve, the accuracy values of the three models can be extracted. It can be seen from the curve that the accuracy of ResNet50 is 0.947. The accuracy of ResNet50 has a significant improvement in the initial stage. After about 15 epochs, the accuracy is close to saturation, gradually leveling off, and basically maintaining above 0.93. EfficientNet v2s has an accuracy of 0.955. The accuracy of the EfficientNet v2s model improves quickly from the beginning, exceeding 0.90 after about 10 epochs, then reaching the peak at nearly 12 epochs, but fluctuating slightly during the subsequent training process. The accuracy of GD-DAMNet is 0.988, and the accuracy of GD-DAMNet is superior. The accuracy of the initial training is rapidly improved, and the fluctuation is relatively small, and it is almost stable at about 0.97, indicating that the model has good stability and accuracy.

The loss function reflects the difference between the predicted value of the model and the true value. Typically, a smaller loss function value is preferred. The orange curve shows how the loss function changes, and below are the loss values for each model. The loss value of ResNet50 is 0.166, and the loss curve shows a significant downward trend, but the decrease in loss value flattens out after 10 epochs and finally stays at about 0.17. Although the decline is not particularly fast, the loss eventually reaches a low level; the loss value of EfficientNet v2s is 0.153. The loss value of the EfficientNet v2s model decreases rapidly and decreases sharply within the first 4 epochs. Compared with ResNet50, its loss value is lower, indicating that the model performs better in loss function optimisation. The GD-DAMNet loss value is 0.065, which is the lowest loss function value, and the rate of decrease is very fast. The loss value drops to close to the final value of 0.065 within the first 7 epochs, demonstrating excellent convergence.

Training stability can be evaluated by the degree of fluctuation in the accuracy curve and loss function curve. The smaller the fluctuation in the curves, the more stable the model training. The accuracy curve of ResNet50 shows significant fluctuations in the early stages of training but stabilises after approximately 5 epochs. The loss curve exhibits minimal fluctuations and shows an overall gradual downward trend. Therefore, the ResNet50 model exhibits good stability in the later stages of training but performs slightly weaker in the early stages; the accuracy curve of EfficientNet v2s shows minor fluctuations after reaching its peak, while the loss curve remains relatively smooth, indicating that the model exhibits some fluctuations during training but maintains overall stability; The accuracy curve of GD-DAMNet is very stable with almost no noticeable fluctuations, and the loss curve is also stable and decreases rapidly.

The performance of the power-line presence recognition network is comprehensively evaluated through four metrics: Accuracy (ACC), Precision (PRE), Sensitivity/Recall (SEN), and Specificity (SPE). Each model was trained 15 times, and the average and standard deviation of the specific numerical indicators are presented in the Table 3:

From Table 2, the traditional ResNet50 approach (ACC ≈ 90.233%) lags behind other deep learning methods on the power-line presence recognition task; analysis shows that the EfficientNet-v2 model achieves ACC ≈ 93.458% on this task, indicating it is better at capturing voxel-level 3D morphology and local spatial features; the proposed GD-DAMNet attains the highest performance with ACC ≈ 98.895%, suggesting that GD-DAMNet can learn more abstract and complex data representations, which helps capture finer-grained features in power-line data with complex backgrounds.

### 4.3. Simulation Results and Analysis

**Figure 14:**
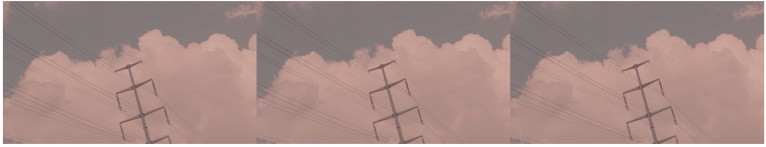
Video image of real-time recognition effect of urban UAV power-line data part.

**Figure 15:**
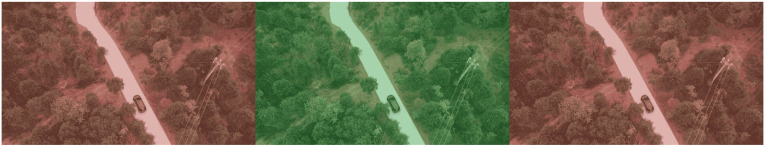
Video image of real-time recognition effect of part 1 of UAV power-line data of Jinkouhe in Leshan, Sichuan.

**Figure 16:**
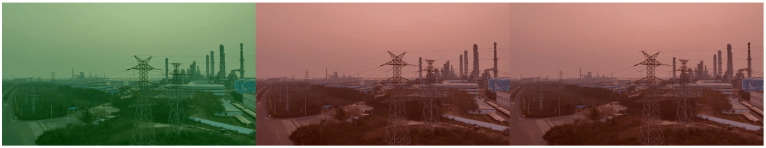
Video image of real-time recognition effect of power lines in the urban high-voltage-line UAV data section.

**Figure 17:**
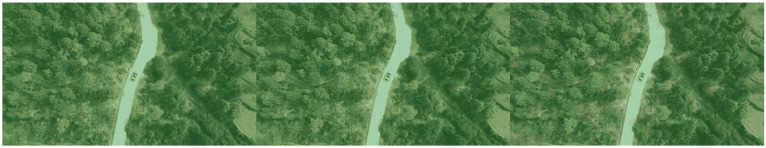
Video image of real-time recognition effect of part 2 of UAV power-line data of Jinkou River in Leshan, Sichuan.

**Figure 18:**
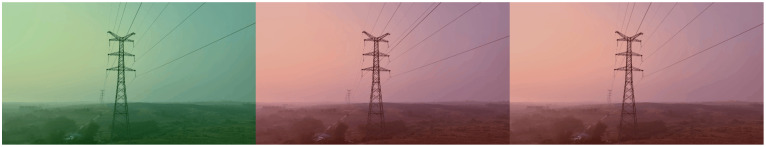
Video image of real-time recognition effect of the UAV power-line data part 1 of the high-voltage line on suburban.

**Figure 19:**
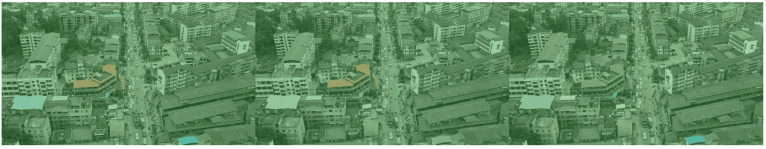
Video image of real-time power-line recognition effect of urban UAV without power-line data part.

**Figure 20:**
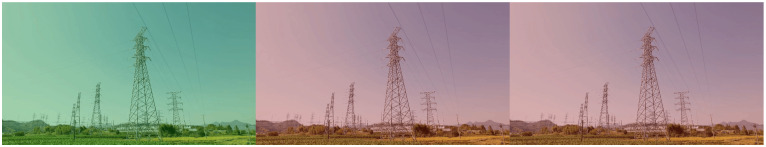
Video image of real-time recognition effect of the UAV power-line data part 2 of the high-voltage line on suburban.

In this study, eight groups of video data are subjected to algorithmic simulation, and, compared with today’s relatively advanced algorithms, such as EfficientNet v2 and ResNet50 network identification, these experimental results show that the proposed GD-DAMNet network is able to achieve high accuracy and high performance in real-time power-line detection tasks and provides an effective solution for practical applications. In Figure 14, Figure 15, Figure 16, Figure 17, Figure 18, Figure 19 and Figure 20, each set of videos shows, from left to right, the outputs of ResNet50, EfficientNet v2, and the proposed GD-DAMNet on the power-line recognition task. Video frames are colour-coded to indicate recognition status: green frames mean no power line was recognised in the current frame, and red frames mean a power line was recognised. The colour coding allows intuitive comparison of the three networks’ recognition response sensitivity, misidentifications, and robustness under different scenes or lighting changes, thereby facilitating evaluation of model performance differences.

Figure 14 shows a real-time recognition video with a duration of 5 s intercepted from the urban UAV power-line data (1 min 19 s) test. In this section, the performance of different networks in recognising urban UAV power-line data can be clearly observed. Among them, The ResNet50 model yields unsatisfactory results. In this 5-s video, the ResNet50 network has more recognition errors or inaccuracies, resulting in a less satisfactory overall recognition result. The EfficientNet v2 and GD-DAMNet networks show higher recognition accuracy. In the entire 10-s video, there are almost no misrecognised frames. This means that they are able to accurately recognise the power lines in each frame, whether in terms of their shape, location, or relationship with the surrounding environment. This high recognition accuracy indicates that the two networks are very robust in recognising urban UAV power-line data.

Figure 15 shows a 5-s real-time recognition video captured in the test of power-line data 1 (32 s) of the Jinkouhe UAV in Leshan, Sichuan. In this video, it can be seen that the recognition efficiency of ResNet50 and EfficientNet v2 networks is not ideal, with a considerable number of misidentifications, while the recognition efficiency of the GD-DAMNet network proposed in this paper is better and has fewer misidentifications and and strong robustness.

Figure 16 shows a 5-s segment of real-time recognition video intercepted from the test of the urban high-voltage-line UAV data (13 s). From the simulation results, the results indicate that the ResNet50 network fails to detect the power lines, while the EfficientNet v2 and GD-DAMNet networks achieve high recognition results, with almost no misrecognition, so these two networks can be practically applied in this case.

Figure 17 shows a 5-s real-time recognition video captured in the test of power-line data 2 (34 s) of the Jinkouhe UAV in Leshan, Sichuan. From the simulation results, it can be seen that the ResNet50 and EfficientNet v2 networks show high misidentification of power lines, while the GD-DAMNet network shows relatively high recognition results with almost no misidentification.

Figure 18 shows a 5-s segment of a real-time identification video intercepted from the test of the suburban high-voltage line UAV power-line data 1 (19 s). From the simulation results, it can be seen that the ResNet50 network exhibits a higher misidentification rate of power lines, while the EfficientNet v2 and GD-DAMNet networks have better recognition accuracy and rarely have misidentification cases.

Figure 19 shows a 5-s segment of a real-time recognition video captured from the test of the urban UAV without power-line data (36 s). From the simulation results, it can be seen that the three networks ResNet50, EfficientNet v2, and GD-DAMNet have excellent performance in the accuracy of no power-line recognition with very few misidentification cases.

Figure 20 shows a 5-s segment of a real-time identification video intercepted from the test of the suburban high-voltage-line UAV data 2 (12 s). From the simulation results, it can be seen that the ResNet50 network does not recognise the power lines, while the EfficientNet v2 and GD-DAMNet networks have high recognition results with almost no misidentification.

To quantify the recognition accuracy of each model, this study converts each video test data into 1000 frames and determines the recognition accuracy by recognising the color, as shown in Table 4:

Table 4 compares the accuracy of the three network models (ResNet50, EfficientNet v2, and GD-DAMNet) for power-line identification on different test datasets. For each model, the “number of unrecognised” and “accuracy”are given. Each line represents the test results of different types of data, including urban UAV power-line data, Leshan Jinkouhe UAV power-line data, urban UAV high-voltage power-line data, and so on. In the urban UAV power-line data test, the number of unrecognised power lines of the ResNet50 network is 579, with an accuracy of 0.421, which indicates that the recognition ability of the ResNet50 model is weak on this kind of data, and over half of the power lines are not recognised. In the test of Leshan Jinkouhe UAV power-line data, the number of unrecognised wires is 293, and the accuracy is 0.707, which is an improvement, but there are still a considerable number of wires unrecognised. Especially in the urban UAV electric high-voltage power-line data and the suburban high-voltage power-line UAV data2, the number of unrecognised power lines reaches 1000, with an accuracy of 0, which means that ResNet50 is unable to recognise power lines at all in this kind of data. Although ResNet50 shows marginal improvement in other tests, its accuracy remains unsatisfactory; for the EfficientNet v2 network in the urban UAV power-line data test, the number of unrecognised power lines is only 28, and the accuracy is close to perfect, reaching 0.972. In the Leshan Jinkouhe UAV power-line data test, the number of unrecognised power lines is 511, with a lower accuracy of 0.489, which shows that the performance varies greatly in different scenarios. In other data tests, the performance of EfficientNet v2 is relatively poor, e.g., the number of unrecognised data in Leshan Jinkouhe UAV power-line data 1 and suburban high-voltage-line UAV power-line data 1 is large, and the accuracy is not ideal; the number of unrecognised data in the Urban UAV power-line data test of GD-DAMNet is only 14, and the accuracy is 0.923; although the number of unrecognised power lines is slightly higher than that of EfficientNet v2, the overall performance remains excellent with accuracy approaching 1. In the test of Leshan Jinkouhe UAV power-line data 1, the number of unrecognised power lines is 77, and the accuracy is 0.923, although the number of unrecognised power lines is slightly higher than that of other test data and the accuracy is significantly better than that of ResNet50 and EfficientNet v2 networks. Moreover, GD-DAMNet performs very well, with the number of unrecognised power lines being 0 and the accuracy reaching 1, which identifies all power lines completely.

ResNet50 performs weakly on most datasets; especially in some complex cases or the case of mixing power lines and no power lines, the recognition is poor. EfficientNet v2 performs very well on some datasets (e.g., urban UAV power-line data), but its performance fluctuates on other datasets; GD-DAMNet has the best performance in general, especially in terms of accuracy, which is very close to 1 on almost all datasets, proving its strong adaptability in the power-line recognition task.

## 5. Discussion

This study evaluates the performance of ResNet50, EfficientNet v2s, and GD-DAMNet models in real-time power-line recognition tasks via a series of experimental trials. The accuracy of ResNet50 is 0.947, which exhibits significant improvement during the initial phase, and approaches saturation and stabilisation after about 15 epochs, basically remaining above 0.93. The accuracy of EfficientNet v2s is 0.955, which improves faster from the beginning, exceeds 0.90 after about 10 epochs, and reaches a peak at about 12 epochs but fluctuates afterwards. The accuracy of GD-DAMNet is 0.988, which improves rapidly and fluctuates little in the early stage of training and stabilises at approximately 0.97, demonstrating robust stability and high accuracy. In multiple tests on video data, such as urban UAV power-line data, Sichuan Leshan Jinkouhe UAV power-line data, and urban high-voltage-line UAV data, the ResNet50 network does not perform well in most cases, and there are a lot of misrecognition or nonrecognition cases. The EfficientNet v2 network performs poorly on some of the data tests (e.g., Leshan Jinkouhe UAV power-line data), but the recognition accuracy is higher on some data (e.g., urban UAV power-line data). The GD-DAMNet network maintains relatively stable and high recognition accuracy under various complex conditions, such as different weather, illumination, shooting angle, and interference factors, with fewer false recognitions and higher robustness. The performance of ResNet50 network varies greatly on different test datasets, and it demonstrates limited capability on urban UAV power-line data and fails to recognise power lines on some data (e.g., urban UAV high-voltage power-line data and suburban high-voltage power-line UAV data 2). The EfficientNet v2 network achieves satisfactory performance well on the urban UAV power-line data, but its performance exhibits considerable variation on tests such as the Leshan Jinkouhe UAV power-line data, where the number of unrecognised power lines is large and the accuracy is unsatisfactory. The GD-DAMNet network delivers strong performance in all datasets; although there are more unrecognised power lines in the Leshan Jinkouhe UAV power-line data than in other tests, the accuracy is significantly better than that of the ResNet50 and EfficientNet v2 networks, and all the power lines can be recognised completely in some datasets. The improved GD-DAMNet network based on the DAM demonstrates superior performance over ResNet50 and EfficientNet v2 networks in the real-time power-line recognition task, both in the training results and the simulation experimental results, and is able to realise high accuracy and high performance, which provides an effective solution for practical applications.

## 6. Conclusions

With the widespread application of UAV technology, the risk of in-flight collisions has become a significant concern with obstacles such as power lines during flight. Traditional power-line recognition methods suffer from several limitations, such as low efficiency of manual inspections and high safety risks. This study proposes a novel MambaDN-GhostNet knowledge distillation dual-attention mechanism CNN for real-time UAV power-line recognition, the GD-DAMNet network model, to enhance recognition accuracy and real-time performance, ensuring safe UAV flight. Model performance was evaluated by observing changes in accuracy and loss function values during training, as well as power-line recognition accuracy across multiple video datasets. In tests on multiple video datasets (e.g., UAV power-line data from urban areas and Leshan Jinkouhe), the ResNet50 network demonstrated poor performance, with numerous misidentifications or unidentified cases; the EfficientNet v2 network showed limited capability on other datasets but achieved high recognition accuracy in others; the GD-DAMNet network maintains relatively stable high recognition accuracy in various complex scenarios, with fewer misidentifications and strong robustness. By converting video test data into 1000 frames and determining recognition accuracy based on identified colours, the GD-DAMNet network demonstrated the best overall performance, with near-perfect accuracy achieved across all datasets, demonstrating exceptional adaptability in wire recognition tasks. The GD-DAMNet network improved with a dual attention mechanism demonstrates advantages over ResNet50 and EfficientNet v2 networks in both training results and simulation experiments in real-time power-line detection tasks, achieving high accuracy and efficiency and providing an effective solution for real-time power-line detection by UAVs.

Although the dual attention mechanism has significantly improved model performance, further optimisation may yield even better results. Other attention mechanisms, such as self-attention or multi-head attention [40], could be considered to further enhance the model’s recognition capability and robustness. Although the current dataset covers a variety of scenarios, it may still have certain limitations. In the future, the dataset scale can be expanded to include more power-line types and samples from complex environments, thereby further enhancing the model’s generalisation capability and adaptability. In practical applications, changes in the environment and power lines may lead to a decline in model performance. Future research can explore adaptive learning algorithms to enable the model to update and adjust online, thereby maintaining efficient recognition performance.

## Figures and Tables

**Table 1:** Information on UAV data collection.

Date	Time	Location	Flight Altitude	Weather
11October 2022	8.00–10.00	High-tech Zone, Leshan, Sichuan	100 m	Sunny day
16 October 2022	8.00–10.00	Gandhara, Leshan, Sichuan	70 m	Heavy fog
19 October 2022	14.00–16.00	Jinkouhe, Leshan, Sichuan	120 m	Sunny day
Web-ordered UAV imaging data	-	Shooting in multiple locations both at home and abroad	Dynamic height	Cloudy, sunny, evening, etc.

**Table 2:** DAMNet network architecture.

Stage	Operator	Stride	Resolution	#Channels	#Layers
-	Input images	-	300 × 300	3	1
0	Conv, k5 × 5	2	150 × 150	24	1
1	CBLSs-Conv1, k3 × 3	1	150 × 150	24	1
2	CBLS-Conv4, k3 × 3	2	75 × 75	48	1
3	CBLSs-Conv4, k3 × 3	2	38 × 38	64	1
4	LSCB-Conv4, k5 × 5	2	19 × 19	128	1
5	LSCBs-Conv6, k5 × 5	1	15 × 15	160	4
6	LSCB-Conv6, k3 × 3	1	7 × 7	256	2
7	LSCB-Conv6, k3 × 3	1	3 × 3	1280	1
8	Conv, k1 × 1 and Pooling and FC	-	-	1280	1

**Table 3:** Model comparison for power-line presence recognition task.

Method	ACC	PRE	SEN	SPE
ResNet50	90.233 ± 2.146	84.582 ± 1.631	86.823 ± 0.927	84.988 ± 0.836
EfficientNet v2	93.458 ± 1.631	89.569 ± 1.215	92.221 ± 0.542	91.060 ± 0.763
GD-DAMNet (Ours)	98.695 ± 0.359	97.505 ± 0.468	96.919 ± 0.471	98.184 ± 0.387

**Table 4:** Comparison of power-line recognition accuracy of three network models for each test data.

Network Model	ResNet50		EfficientNet v2		GD-DAMNet	
Type of Test Data	Number of Unrecognised	Accuracy	Number of Unrecognised	Accuracy	Number of Unrecognised	Accuracy
Urban UAV power-line data	579	0.421	28	0.972	14	0.986
Leshan Jinkouhe UAV power-line data 1	293	0.707	511	0.489	77	0.923
Urban UAV high-voltage power-line data	1000	0	0	1	0	1
Leshan Jinkouhe UAV power-line data 2	337	0.663	327	0.673	10	0.990
Suburban high-voltage-line UAV power-line data 1	352	0.648	0	1	0	1
Urban UAV without power-line data	17	0.983	0	1	0	1
Suburban high-voltage-line UAV power-line data 2	1000	0	0	1	0	1

## Data Availability

The data presented in this study are available on request from the corresponding author.
